# Supplementary material for: The influence of UV-visible light, microwave radiation, argon laser, and heating and aging processes on silicone oil utilized as intravitreal implants: Experimental exposure with clinical correlation
Source: PLoS One. 2024 Dec 31;19(12):e0316212. doi: 10.1371/journal.pone.0316212 (PMC11687662; doi:10.1371/journal.pone.0316212)
Supplement: Attachment — Submitted filename: Response_letter_final.docx [file pone.0316212.s004.docx]

Academic Editor

PLOS ONE

Dear editor,

My self and the co-authors are pleased to resubmit the revised version of the manuscript entitled ‘The influence of UV-visible light, microwave radiation, argon laser, and heating and aging processes on silicone oil utilized as intravitreal implants: Experimental exposure with clinical correlation, ID: PONE-D-24-26615R1” to be considered for publication in PLOS ONE. The revised manuscript takes into consideration all editorial comments. Kindly find below a point-by-point response to those comments, along with an uploaded copy marked with MS track changes indicating changes to the manuscript.

Again and again, many thanks for our respectful editor and the editorial staff for their efforts. Your efforts are much appreciated and valuable and will be kept in heart

**Editorial comments**

Thank you so much for your efforts and time in improving this manuscript.

“Thanks for submitting your revised manuscript. Unfortunately professional English language editing was not performed. The English language still needs extensive editing and correction of spelling mistakes. For example, one of your subtitles reads “partients and design”. I suggest to use a professional English language editor to improve the style of the manuscript.”

**Response**: Thank you so much for your efforts and the comments. We have sent the manuscript for a professional English language editing service and we have uploaded the certificate. Thank you again

**Journal Requirements**

1. Please review your reference list to ensure that it is complete and correct. If you have cited papers that have been retracted, please include the rationale for doing so in the manuscript text, or remove these references and replace them with relevant current references. Any changes to the reference list should be mentioned in the rebuttal letter that accompanies your revised manuscript. If you need to cite a retracted article, indicate the article’s retracted status in the References list and also include a citation and full reference for the retraction notice.

**Response**: Thanks so much. We have revised and modified the reference list. Furthermore, to cooperate with your efforts, we have included two references lists, one without software and one arranged with the software.

Again, we thank the editor and reviewers for their constructive comments. We hope that we have sufficiently addressed their concerns.

Sincerely,

Authors
